# Measuring dementia incidence within a cohort of 267,153 older Australians using routinely collected linked administrative data

**DOI:** 10.1038/s41598-020-65273-w

**Published:** 2020-05-29

**Authors:** Heidi J. Welberry, Henry Brodaty, Benjumin Hsu, Sebastiano Barbieri, Louisa R. Jorm

**Affiliations:** 1 Centre for Big Data Research in Health, University of New South Wales, Sydney, New South Wales Australia; 2 Centre for Healthy Brain Ageing, School of Psychiatry, University of New South Wales, Sydney, New South Wales Australia; 3 Dementia Centre for Research Collaboration, School of Psychiatry, University of New South Wales, Sydney, New South Wales Australia

**Keywords:** Dementia, Risk factors, Epidemiology

## Abstract

To estimate dementia incidence rates using Australian administrative datasets and compare the characteristics of people identified with dementia across different datasets. This data linkage study used a cohort of 267,153 from the Australian 45 and Up Study. Participants completed a survey in 2006–2009 and subsequent dementia was identified through pharmaceutical claims, hospitalisations, aged care eligibility assessments, care needs at residential aged care entry and death certificates. Age-specific, and age-standardised incidence rates, incidence rate ratios and survival from first dementia diagnosis were estimated. Estimated age-standardised dementia incidence rates using all linked datasets was 16.8 cases per 1000 person years for people aged 65+. Comparing incidence rates to the global published rates suggested 77% of cases were identified but this varied by age with highest coverage among those aged 80–84 years (92%). Incidence rate ratios were inconsistent across datasets for: sex, socio-economic disadvantage, size of support network, marital status, functional limitations and diabetes. Median survival from first dementia diagnosis ranged from 1.80 years in the care needs dataset to 3.74 years in the pharmaceutical claims dataset. Characteristics of people identified with dementia in different administrative datasets reflect the factors that drive interaction with specific services; this may introduce bias in observational studies using a single data-source to identify dementia.

## Introduction

Routinely-collected linked administrative data are increasingly being used to monitor endpoints in observational studies and clinical trials^1^. Dementia prevention studies may benefit from this approach due to the long time-frame required to study risk factors in this population^2^. Within a research setting, maintaining contact with people in older age groups, particularly as they develop cognitive impairment or dementia is often not viable. Administrative data have the potential to increase power within studies by improving completeness of follow-up, and to reduce bias by avoiding the issue of differential drop-out due to cognitive impairment^3^. However, there are also potential limitations to using administrative databases for detecting dementia.

Administrative health data are those generated routinely via a person’s interaction with the health system. They may include records of hospitalisation, physician visits, entry to long term care or dispensing of pharmaceuticals. They are generally collected with payment rather than research in mind, but there is often a degree of data curation that increases accuracy or adds value such as through coding of diseases and medical conditions. As such, they can be a valuable resource for research^4^. When using such data to measure endpoints in trials or cohort studies it is important to consider both whether the cases identified have the disease of interest (usually indicated by high positive predictive value (PPV)) and the proportion of total cases that are detected (sensitivity). A recent systematic review examined the accuracy of dementia coding within routinely collected administrative datasets against expert-derived reference standards^1^. They found that a high proportion of dementia cases detected within administrative datasets did truly have dementia (PPV of 70–90%)^1^. However, sensitivity was found to be poor with only 30–50% of true cases detected^1^.

One approach to increasing sensitivity is to use multiple administrative datasets to increase the likelihood of dementia detection^5^. The primary care physician is usually the first health professional consulted in Australia regarding dementia symptoms and then a referral to a specialist would be made to confirm a diagnosis^6^. Currently in Australia routinely collected primary care and outpatient specialist claims do not include diagnostic codes. Nevertheless, there are multiple sources of data available that provide good chances of detecting dementia. These include hospital records, pharmaceutical claims, long term care assessments and cause of death records. A recent study using the Australian Longitudinal Study on Women’s Health demonstrated the feasibility of using these combined data sources to estimate dementia prevalence and incidence^5^. Through use of Capture-Recapture techniques, they estimated that these combined datasets detected approximately 80% of all underlying cases in their population.

However, it does not necessarily follow that it is appropriate to use such combined data to measure an endpoint in a study or trial. It is analogous to running a trial where outcomes are assessed using different methods and at varying follow-up times for different subsets of participants. If a person has an equal chance of appearing within any administrative database and the chance of being captured within a database does not vary with time, age or other factors, then it is unlikely to have any impact on the outcome. However, this is not the case with administrative health data. Hospital admissions are influenced by age and sex as well as a range of chronic diseases and risk factors^7^, only a small subset of eligible people are prescribed dementia-specific pharmaceuticals^8^, accessing long-term care is often restricted by age and influenced by social factors^9,10^ and recording of dementia on a death certificate can be influenced by age and other co-morbidities^11^.

It is possible therefore that different results may be obtained dependent on which combinations of administrative datasets are used. Johnson *et al*. demonstrated that using different combinations of datasets to identify patients at high-risk of hospitalisation will identify different sub-groups of patients^12^. Similarly, Lujic *et al*. showed that there were differences in characteristics of those classified as having multi-morbidity when comparing self-report data to hospitalisations, pharmaceutical claims or a combination of the three^13^.

While various studies have provided insight into the detection of dementia using administrative data^14–20^, nearly all have focussed on quantifying the PPV or sensitivity of a data source or other validation measures such as specificity. Østbye *et al*.^20^ examined bias based on socio-demographic variation in diagnoses but no study has examined in-depth how dementia detection within administrative data may vary by health-related characteristics. There has also been no study to our knowledge using Australian data that has examined potential bias in dementia detection using multiple administrative data sources.

The aim of this study is to provide guidance on the current usefulness of multiple linked administrative data in detecting dementia. We investigated: (i) estimated age-specific dementia incidence rates based on multiple-linked datasets versus individual datasets to establish whether incidence patterns align with those found in other cohort studies which used clinical diagnoses to establish dementia; (ii) individual characteristics associated with relative dementia incidence rates in each dataset to investigate potential biases; and (iii) survival from first date of dementia diagnosis by source of dementia detection to assess any differences in timing of detection along the trajectory of dementia progression.

## Results

Of the 267,153 people who completed the 45 and Up Study baseline survey in 2006–2009, there were 261,910 alive two years later and eligible to enter this study. Of these, 69 were excluded due to probable data linkage errors, 2535 who had a recorded dementia diagnosis prior to the study entry date, and 4280 who held Department of Veteran’s Affairs Gold Card health insurance. There were 255,026 people included in the final cohort. The mean age of the cohort at entry (2 years after baseline survey) was 64.1 years (SD = 10.9) and 46% were male.

The average duration of follow-up was 4.2 years (range 0.003–6.5 years) and 20,812 people died within the follow-up period. There were 5945 unique cases of dementia identified across the 5 administrative datasets and 1062980 years of follow up, resulting in an estimated crude incidence rate for the cohort of 5.6 cases per 1000 person years. Of the 5945 cases, 1837 were identified in pharmaceutical claims data (31%), 3054 in hospital inpatient records (51%), 2833 in aged care assessments (48%), 2767 in the aged care funding instrument (47%) and 824 in death certificates (14%). Almost half the cases (2844 or 48%) were identified in only one dataset, 1484 (25%) in two, 1049 (18%) in three, 484 (8%) in four, and 84 cases (1%) were found in all five datasets. Of the 2844 identified in only one dataset, 660 (23%) were within pharmaceutical claims, 913 in hospital records (32%), 489 in aged care assessments (17%), 672 in the ACFI (24%), and 110 in death certificates (4%).

**Figure 1:**
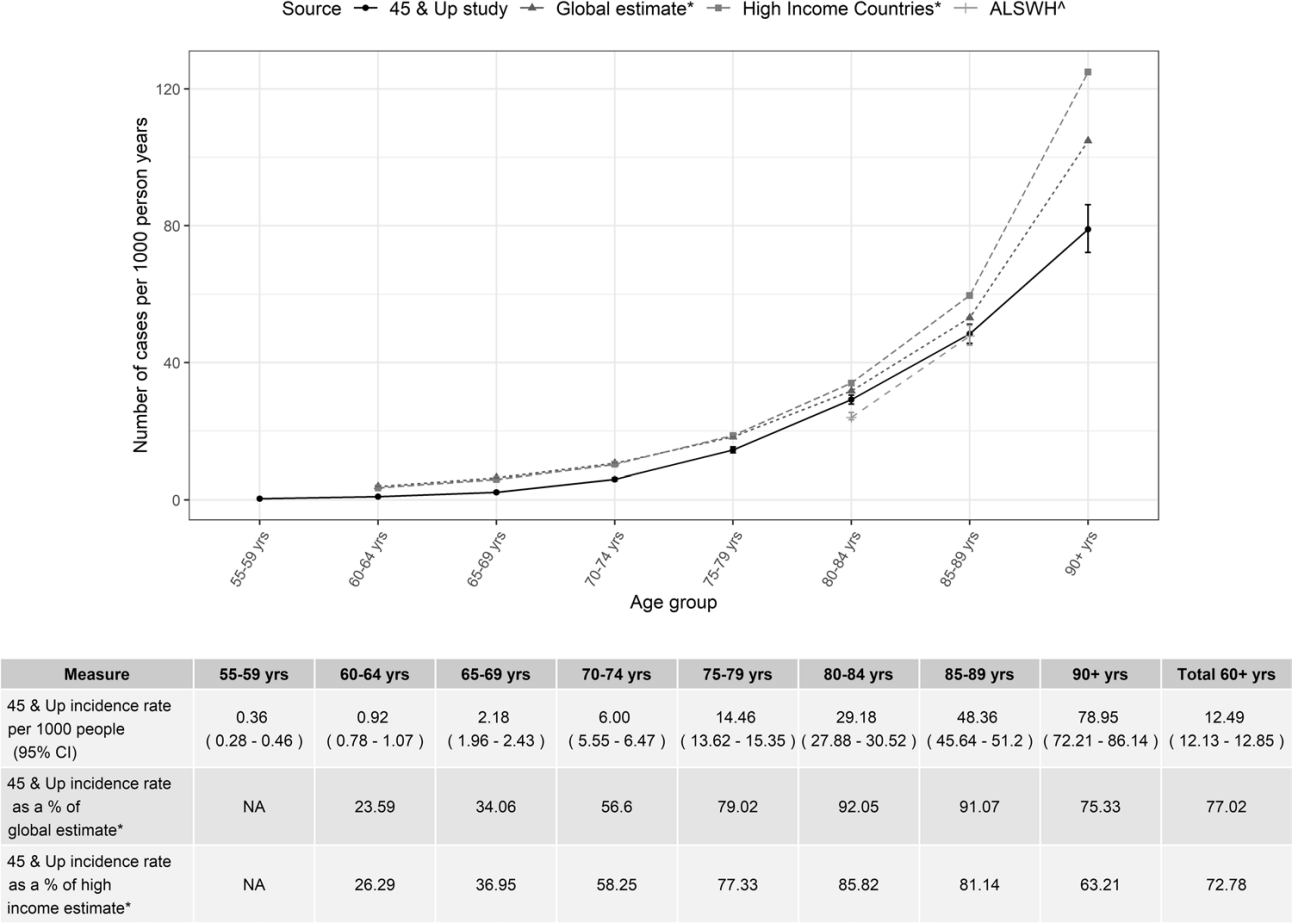
Age-specific dementia incidence rates compared to estimates from other studies. Rates are compared to: *The global and high-income rates from the Global Impact of Dementia study^22^ and to ^The Australian Longitudinal Study on Women’s Health (ALSWH)^5^.

**Figure 2:**
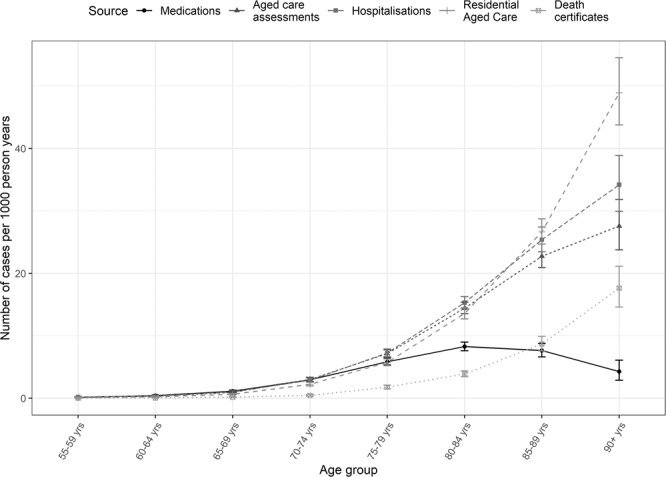
Age-specific dementia estimates by individual data sources. Coverage in each dataset varies with age. Medications data may be poor at detecting dementia in the older age groups whereas death certificates are poorer in the younger age groups.

Age-specific dementia incidence rates are presented in Fig. 1 and compared to estimates derived from other studies. Estimated incidence rates rose from 0.4 cases per 1000 person years in the 55–59-year group (95%CI: 0.3–0.5) to 79 cases per 1000 person years (95%CI: 72–86) in those aged 90 years or older. Figure 2 presents the age specific rates calculated using each of the five data sources individually. Incidence rates increased with age across all data sources except for pharmaceutical claims which showed a peak within the 80–84-year age group and then a decline for those aged 85 years and older. The overall age-standardised rate of dementia incidence was 9.68 (95%CI: 9.40–9.95), 12.49 (95%CI: 12.13–12.85) and 16.93 (95%CI: 16.44–17.42) cases per 1000 person years for those aged 55 years or older, 60 years or older and 65 years or older respectively.

Tables 1, 2, 3 present case characteristics based on the datasets in which they were detected. The mean age at study entry (two years following the 45 and Up baseline survey) of cases based on each dataset was 75.4 years in pharmaceutical claims, 78.6 years in aged care assessments, 78.7 years in hospitalisations, 80.2 years in the ACFI and 81.2 years in death certificates. More females were represented within pharmaceutical claims and the ACFI and fewer in aged care assessments, hospitalisations and death certificates. Marital status varied considerably across data sources ranging from 71% of those within pharmaceutical claims data having been married or partnered at baseline compared to 61% for aged care assessments, 58% hospitalisations, 55% death certificates and 49% for the ACFI.

**Table 1:** Socio-demographic characteristics of dementia cases flagged within each administrative dataset.

Level	Pharmaceutical claims	Aged Care assessments	Hospitalisations	Residential aged care funding instrument	Death certificates	Combined
	n(%)	n(%)	n(%)	n(%)	n(%)	n(%)
Number of cases	1837	2833	3054	2767	824	5945
**Died during study period**						
No	1206 (65.7)	1264 (44.6)	1063 (34.8)	920 (33.2)	0 (0.0)	2692 (45.3)
Yes	631 (34.3)	1569 (55.4)	1991 (65.2)	1847 (66.8)	824 (100.0)	3253 (54.7)
Age at study commencement – Mean (SD)	75.38 (7.36)	78.58 (7.32)	78.68 (7.76)	80.20 (7.40)	81.20 (7.19)	78.45 (8.02)
**Sex**						
Male	907 (49.4)	1452 (51.3)	1642 (53.8)	1306 (47.2)	452 (54.9)	3033 (51.0)
Female	930 (50.6)	1381 (48.7)	1412 (46.2)	1461 (52.8)	372 (45.1)	2912 (49.0)
**Marital Status**						
Single	52 (2.8)	138 (4.9)	188 (6.2)	201 (7.3)	53 (6.4)	350 (5.9)
Married/Partner	1312 (71.4)	1718 (60.6)	1778 (58.2)	1368 (49.4)	452 (54.9)	3455 (58.1)
Widowed/divorced/separated	461 (25.1)	964 (34.0)	1064 (34.8)	1176 (42.5)	315 (38.2)	2095 (35.2)
Missing	12 (0.7)	13 (0.5)	24 (0.8)	22 (0.8)	4 (0.5)	45 (0.8)
**Education**						
Did not complete school	772 (42.0)	1239 (43.7)	1398 (45.8)	1292 (46.7)	382 (46.4)	2637 (44.4)
High school/trade	753 (41.0)	1097 (38.7)	1144 (37.5)	1003 (36.2)	298 (36.2)	2280 (38.4)
University or higher	257 (14.0)	373 (13.2)	382 (12.5)	348 (12.6)	100 (12.1)	780 (13.1)
Missing/invalid	55 (3.0)	124 (4.4)	130 (4.3)	124 (4.5)	44 (5.3)	248 (4.2)
**Household income**						
<$10,000	185 (10.1)	313 (11.0)	364 (11.9)	319 (11.5)	91 (11.0)	677 (11.4)
$10,000–$29,999	574 (31.2)	973 (34.3)	1058 (34.6)	1010 (36.5)	283 (34.3)	2058 (34.6)
$30,000–$49,999	259 (14.1)	324 (11.4)	340 (11.1)	252 (9.1)	82 (10.0)	661 (11.1)
$50,000–$69,999	96 (5.2)	116 (4.1)	108 (3.5)	88 (3.2)	31 (3.8)	235 (4.0)
$70,000 or more	106 (5.8)	115 (4.1)	118 (3.9)	97 (3.5)	27 (3.3)	259 (4.4)
Not specified	390 (21.2)	558 (19.7)	621 (20.3)	535 (19.3)	171 (20.8)	1189 (20.0)
Missing	227 (12.4)	434 (15.3)	445 (14.6)	466 (16.8)	139 (16.9)	866 (14.6)
**Remoteness Area**						
Major Cities	1056 (57.5)	1770 (62.5)	1809 (59.2)	1690 (61.1)	523 (63.5)	3551 (59.7)
Inner Regional	599 (32.6)	809 (28.6)	925 (30.3)	825 (29.8)	229 (27.8)	1803 (30.3)
Outer Regional/Remote/Very Remote	160 (8.7)	217 (7.7)	274 (9.0)	220 (8.0)	62 (7.5)	506 (8.5)
Missing	22 (1.2)	37 (1.3)	46 (1.5)	32 (1.2)	10 (1.2)	85 (1.4)
**Index of Relative Socioeconomic Disadvantage**						
Q1 Most disadvantaged	408 (22.2)	720 (25.4)	856 (28.0)	793 (28.7)	214 (26.0)	1573 (26.5)
Q2	401 (21.8)	591 (20.9)	675 (22.1)	599 (21.6)	177 (21.5)	1293 (21.7)
Q3	323 (17.6)	518 (18.3)	519 (17.0)	479 (17.3)	134 (16.3)	1037 (17.4)
Q4	284 (15.5)	453 (16.0)	463 (15.2)	393 (14.2)	139 (16.9)	892 (15.0)
Q5 Least disadvantaged	378 (20.6)	492 (17.4)	472 (15.5)	448 (16.2)	140 (17.0)	1011 (17.0)
Missing/invalid	43 (2.3)	59 (2.1)	69 (2.3)	55 (2.0)	20 (2.4)	139 (2.3)
**Country of Birth**						
Australian born	1318 (71.7)	2010 (70.9)	2143 (70.2)	1951 (70.5)	557 (67.6)	4169 (70.1)
Born overseas	493 (26.8)	780 (27.5)	853 (27.9)	770 (27.8)	257 (31.2)	1678 (28.2)
Missing	26 (1.4)	43 (1.5)	58 (1.9)	46 (1.7)	10 (1.2)	98 (1.6)
**Language Spoken at Home**						
English only	1665 (90.6)	2545 (89.8)	2726 (89.3)	2501 (90.4)	730 (88.6)	5331 (89.7)
Other language	172 (9.4)	288 (10.2)	328 (10.7)	266 (9.6)	94 (11.4)	614 (10.3)
Missing	0 (0.0)	0 (0.0)	0 (0.0)	0 (0.0)	0 (0.0)	0 (0.0)
**Number of people can depend on**						
Zero	75 (4.1)	160 (5.6)	200 (6.5)	179 (6.5)	53 (6.4)	370 (6.2)
1–4	730 (39.7)	1262 (44.5)	1380 (45.2)	1295 (46.8)	388 (47.1)	2636 (44.3)
5–10	648 (35.3)	898 (31.7)	906 (29.7)	804 (29.1)	224 (27.2)	1836 (30.9)
11+	250 (13.6)	272 (9.6)	289 (9.5)	229 (8.3)	68 (8.3)	588 (9.9)
Missing/Invalid	134 (7.3)	241 (8.5)	279 (9.1)	260 (9.4)	91 (11.0)	515 (8.7)

**Table 2:** Health risk factor characteristics of dementia cases flagged within each administrative dataset.

Level	Pharmaceutical claims	Aged Care assessments	Hospitalisations	Residential aged care funding instrument	Death certificates	Combined
	n(%)	n(%)	n(%)	n(%)	n(%)	n(%)
Never smoked	1132 (61.6)	1692 (59.7)	1745 (57.1)	1700 (61.4)	493 (59.8)	3541 (59.6)
Past smoker	644 (35.1)	1013 (35.8)	1137 (37.2)	941 (34.0)	289 (35.1)	2113 (35.5)
Current smoker	50 (2.7)	108 (3.8)	143 (4.7)	110 (4.0)	32 (3.9)	245 (4.1)
Missing/invalid	11 (0.6)	20 (0.7)	29 (0.9)	16 (0.6)	10 (1.2)	46 (0.8)
**Number of drinks per week**						
zero	640 (34.8)	1125 (39.7)	1292 (42.3)	1201 (43.4)	359 (43.6)	2430 (40.9)
1–4	313 (17.0)	453 (16.0)	465 (15.2)	431 (15.6)	136 (16.5)	919 (15.5)
5–7	335 (18.2)	460 (16.2)	456 (14.9)	423 (15.3)	131 (15.9)	932 (15.7)
8–14	287 (15.6)	369 (13.0)	391 (12.8)	335 (12.1)	95 (11.5)	787 (13.2)
15+	190 (10.3)	258 (9.1)	276 (9.0)	207 (7.5)	59 (7.2)	551 (9.3)
missing	72 (3.9)	168 (5.9)	174 (5.7)	170 (6.1)	44 (5.3)	326 (5.5)
**BMI category**						
underweight	243 (13.2)	445 (15.7)	453 (14.8)	449 (16.2)	135 (16.4)	869 (14.6)
normal weight	771 (42.0)	1174 (41.4)	1258 (41.2)	1149 (41.5)	372 (45.1)	2453 (41.3)
overweight	610 (33.2)	863 (30.5)	924 (30.3)	806 (29.1)	218 (26.5)	1826 (30.7)
obese	191 (10.4)	321 (11.3)	388 (12.7)	330 (11.9)	90 (10.9)	734 (12.3)
missing	22 (1.2)	30 (1.1)	31 (1.0)	33 (1.2)	9 (1.1)	63 (1.1)
**Physical activity**						
Does not meet guidelines	855 (46.5)	1564 (55.2)	1757 (57.5)	1676 (60.6)	507 (61.5)	3310 (55.7)
Meets guidelines	982 (53.5)	1269 (44.8)	1297 (42.5)	1091 (39.4)	317 (38.5)	2635 (44.3)
**Physical Limitations (SF36)**						
No Limitations	262 (14.3)	287 (10.1)	261 (8.5)	225 (8.1)	65 (7.9)	566 (9.5)
Minor Limitation	405 (22.0)	460 (16.2)	392 (12.8)	335 (12.1)	94 (11.4)	857 (14.4)
Moderate Limitation	510 (27.8)	780 (27.5)	770 (25.2)	684 (24.7)	194 (23.5)	1541 (25.9)
Severe Limitation	364 (19.8)	803 (28.3)	1071 (35.1)	989 (35.7)	325 (39.4)	1934 (32.5)
Missing	296 (16.1)	503 (17.8)	560 (18.3)	534 (19.3)	146 (17.7)	1047 (17.6)
**Psychological Distress (K10)**						
Low	1377 (75.0)	2090 (73.8)	2183 (71.5)	2004 (72.4)	582 (70.6)	4325 (72.8)
Moderate	247 (13.4)	357 (12.6)	410 (13.4)	356 (12.9)	97 (11.8)	783 (13.2)
High	70 (3.8)	136 (4.8)	171 (5.6)	130 (4.7)	51 (6.2)	297 (5.0)
Very High	27 (1.5)	48 (1.7)	71 (2.3)	49 (1.8)	11 (1.3)	114 (1.9)
Missing	116 (6.3)	202 (7.1)	219 (7.2)	228 (8.2)	83 (10.1)	426 (7.2)

**Table 3:** Health status characteristics of dementia cases flagged within each administrative dataset.

Level	Pharmaceutical claims	Aged Care assessments	Hospitalisations	Residential aged care funding instrument	Death certificates	Combined
	n(%)	n(%)	n(%)	n(%)	n(%)	n(%)
**Cancer**						
No	1405 (76.5)	2151 (75.9)	2327 (76.2)	2085 (75.4)	638 (77.4)	4547 (76.5)
Yes	432 (23.5)	682 (24.1)	727 (23.8)	682 (24.6)	186 (22.6)	1398 (23.5)
**Diabetes**						
No	1637 (89.1)	2434 (85.9)	2556 (83.7)	2343 (84.7)	692 (84.0)	5064 (85.2)
Yes	200 (10.9)	399 (14.1)	498 (16.3)	424 (15.3)	132 (16.0)	881 (14.8)
**Stroke**						
No	1728 (94.1)	2584 (91.2)	2734 (89.5)	2490 (90.0)	737 (89.4)	5376 (90.4)
Yes	109 (5.9)	249 (8.8)	320 (10.5)	277 (10.0)	87 (10.6)	569 (9.6)
**Parkinson’s**						
No	1775 (96.6)	2734 (96.5)	2920 (95.6)	2621 (94.7)	786 (95.4)	5713 (96.1)
Yes	62 (3.4)	99 (3.5)	134 (4.4)	146 (5.3)	38 (4.6)	232 (3.9)
**Depression/Anxiety**						
No	1556 (84.7)	2417 (85.3)	2588 (84.7)	2397 (86.6)	727 (88.2)	5072 (85.3)
Yes	281 (15.3)	416 (14.7)	466 (15.3)	370 (13.4)	97 (11.8)	873 (14.7)
**Heart Disease**						
No	1485 (80.8)	2226 (78.6)	2321 (76.0)	2144 (77.5)	616 (74.8)	4588 (77.2)
Yes	352 (19.2)	607 (21.4)	733 (24.0)	623 (22.5)	208 (25.2)	1357 (22.8)
**Falls**						
No	1277 (69.5)	1778 (62.8)	1814 (59.4)	1618 (58.5)	467 (56.7)	3632 (61.1)
Yes	433 (23.6)	812 (28.7)	971 (31.8)	904 (32.7)	276 (33.5)	1834 (30.8)
missing	127 (6.9)	243 (8.6)	269 (8.8)	245 (8.9)	81 (9.8)	479 (8.1)
**Self-reported Memory**						
Excellent	50 (2.7)	129 (4.6)	171 (5.6)	142 (5.1)	42 (5.1)	306 (5.1)
Very good	250 (13.6)	397 (14.0)	462 (15.1)	380 (13.7)	93 (11.3)	893 (15.0)
Good	641 (34.9)	931 (32.9)	1008 (33.0)	949 (34.3)	250 (30.3)	2020 (34.0)
Fair	656 (35.7)	949 (33.5)	959 (31.4)	871 (31.5)	304 (36.9)	1880 (31.6)
Poor	130 (7.1)	221 (7.8)	234 (7.7)	204 (7.4)	72 (8.7)	427 (7.2)
Missing	110 (6.0)	206 (7.3)	220 (7.2)	221 (8.0)	63 (7.6)	419 (7.0)
**Died during study period**						
No	1206 (65.7)	1264 (44.6)	1063 (34.8)	920 (33.2)	0 (0.0)	2692 (45.3)
Yes	631 (34.3)	1569 (55.4)	1991 (65.2)	1847 (66.8)	824 (100.0)	3253 (54.7)

**Figure 3:**
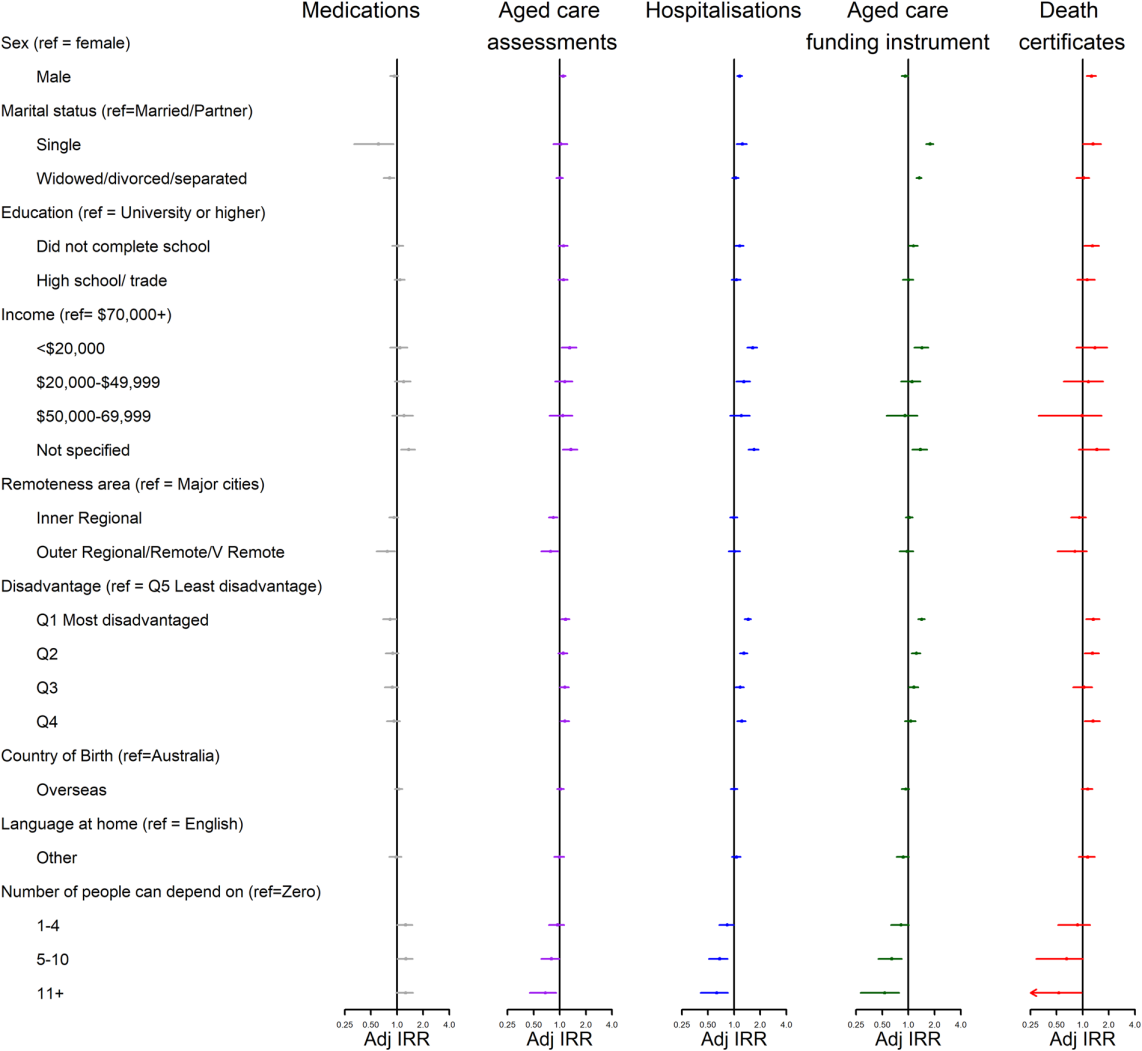
Dementia Incidence Rate Ratios for socio-demographic factors, by administrative data source used to identify dementia. Missing values were present for all variables except sex. They were treated as separate categories for each variable but are excluded from this figure due to small numbers. Adj IRR = Age-adjusted Dementia Incidence Rate Ratio with 95% Confidence Interval”.

**Figure 4:**
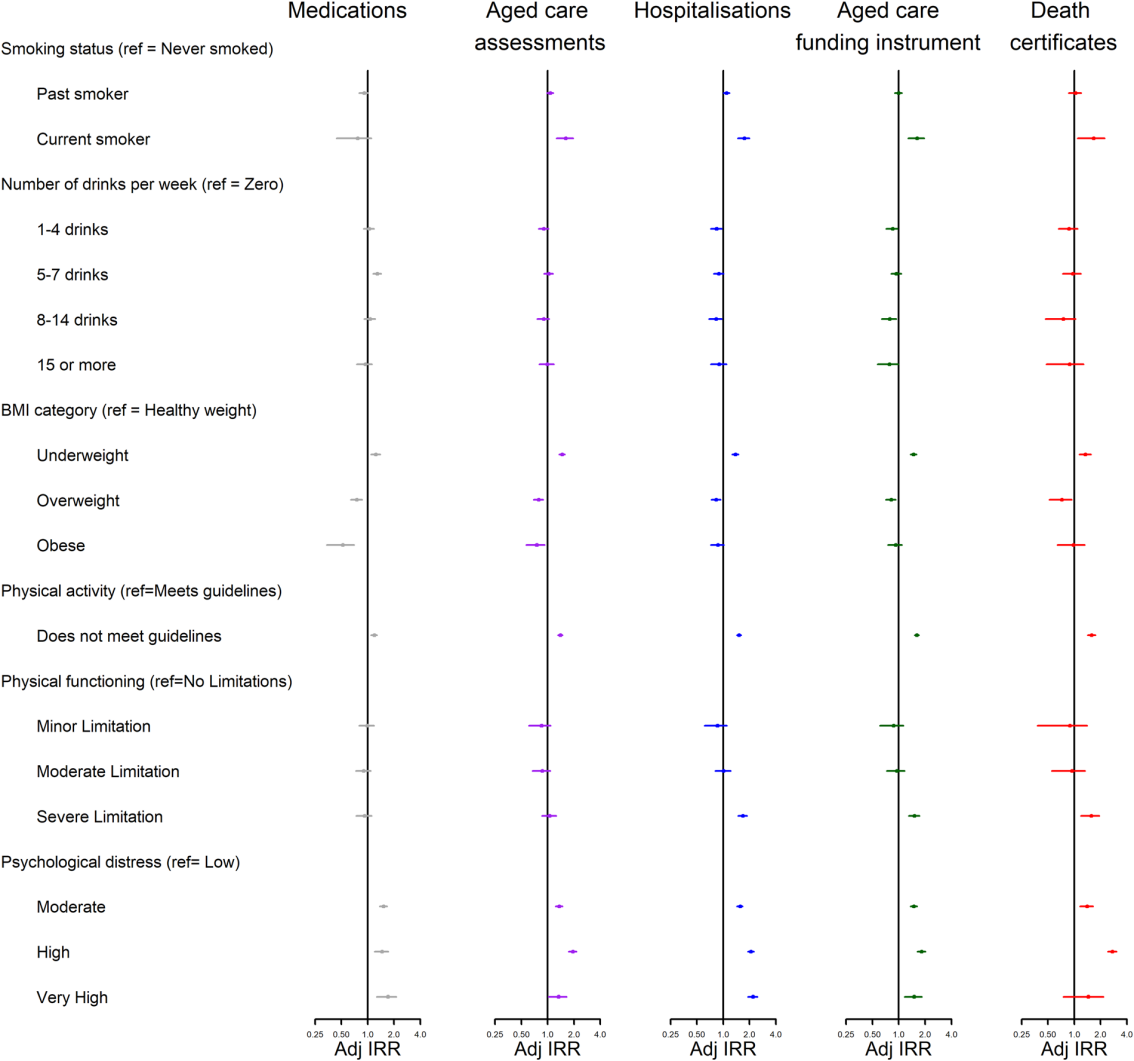
Dementia Incidence Rate Ratios for socio-demographic factors, by administrative data source used to identify dementia. Missing values were present for all variables except sex. They were treated as separate categories for each variable but are excluded from this figure due to small numbers. Adj IRR = Age-adjusted Dementia Incidence Rate Ratio with 95% Confidence Interval”.

**Figure 5:**
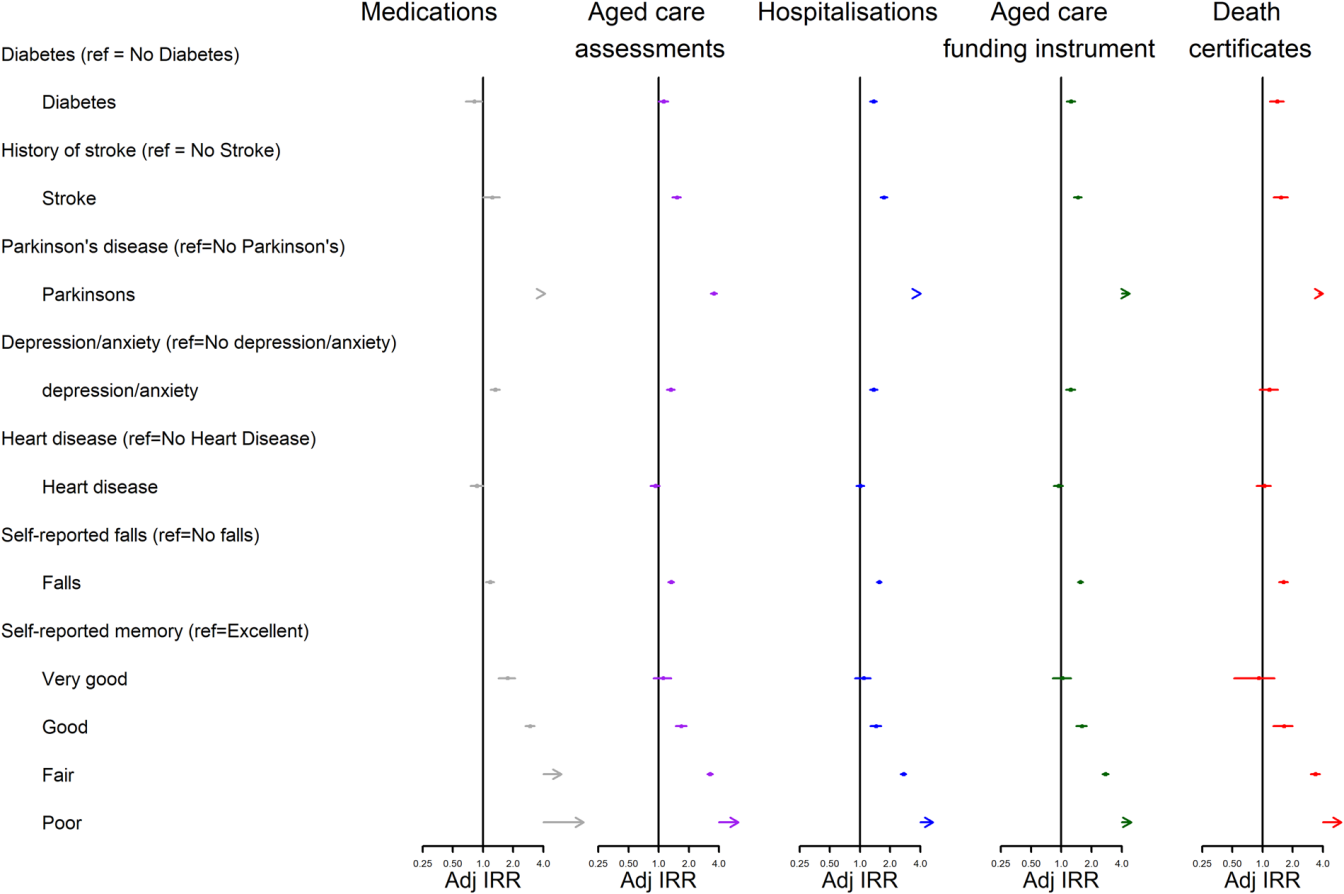
Dementia Incidence Rate Ratios for socio-demographic factors, by administrative data source used to identify dementia. Missing values were present for all variables except sex. They were treated as separate categories for each variable but are excluded from this figure due to small numbers. Adj IRR = Age-adjusted Dementia Incidence Rate Ratio with 95% Confidence Interval”.

Figures 3, 4 and 5 present age-standardised incidence rate ratios (IRR) by data source and baseline characteristics. More detailed results for these figures are provided in Supplementary Table S1. Overall, the pattern of relationships between baseline characteristics and relative dementia incidence rates were similar across datasets but there were inconsistencies in direction of some relationships.

Dementia incidence rates were similar across sexes when using pharmaceutical claims (IRR = 0.93, 95%CI: 0.83–1.03), higher among males when using aged care assessments (IRR = 1.09; 95%CI: 1.01–1.17), hospitalisations (IRR = 1.16; 95%CI: 1.08–1.24) or death certificates (IRR = 1.26; 95%CI: 1.11–1.42) but marginally lower among males when using the ACFI (IRR = 0.92; 95%CI: 0.83–1.00).

In four out of five datasets, people who lived in the most disadvantaged areas had a higher relative incidence of dementia compared to those in the least disadvantaged areas (aged care assessments: IRR = 1.16, 95%CI: 1.04–1.29; hospitalisations: IRR = 1.45, 95%CI: 1.32–1.57; ACFI: IRR = 1.42, 95%CI: 1.29–1.55; death certificates: IRR = 1.32, 95%CI: 1.09–1.56). Conversely using pharmaceutical claims, people who lived in the most disadvantaged areas had lower dementia incidence rates (IRR = 0.83, 95%CI: 0.69–0.98).

Having more people who could be depended on was generally associated with a lower dementia incidence rate (aged care assessments (11+ people vs zero): IRR = 0.68, 95%CI: 0.45–0.91; hospitalisations(11+ people vs zero): IRR = 0.63, 95%CI: 0.41–0.85; ACFI (11+ people vs zero): IRR = 0.53, 95%CI: 0.28–0.78; death certificates(11+ people vs zero): IRR = 0.53, 95%CI: 0.09–0.97). This was not the pattern within pharmaceutical claims (11+ people vs zero: IRR = 1.25, 95%CI: 0.98–1.53; 5–10 people vs zero: IRR = 1.26, 95%CI: 1.01–1.52).

Compared to those who were married or partnered, those who were single had a higher dementia incidence rate using hospitalisations (IRR = 1.24, 95%CI: 1.07–1.40) and ACFI (IRR = 1.77, 95%CI: 1.60–1.94), but lower incidence rate when using pharmaceutical claims (IRR = 0.61, 95%CI: 0.32–0.91) and no difference when using aged care assessments (IRR = 1.03, 95%CI: 0.84–1.22) or death certificates (IRR = 1.31, 95%CI: 1.00–1.62).

In three out of five datasets, diabetes was associated with a significantly higher dementia incidence rate (hospitalisations: IRR = 1.37, 95%CI: 1.26–1.47; ACFI: IRR = 1.25, 95%CI: 1.13–1.37; death certificates: IRR = 1.40, 95%CI: 1.18–1.62) and aged care assessments showed a marginally higher incidence rate (IRR = 1.12, 95%CI: 1.00–1.25). Pharmaceutical claims showed the opposite relationship (IRR = 0.82, 95%CI: 0.67–0.98).

Two other variables (smoking status and physical limitations) showed inconsistent patterns across datasets. Being a current smoker compared to a non-smoker was related to an increase in dementia incidence rates within aged care assessments (IRR = 1.61, 95%CI: 1.26–1.91), hospitalisations (IRR = 1.74, 95%CI: 1.47–2.00), the ACFI (IRR = 1.62, 95%CI: 1.28–1.95) and death certificates (IRR = 1.66, 95%CI: 1.09–2.23), but was associated with a non-significant reduction in dementia incidence rates within pharmaceutical claims (IRR = 0.77, 95%CI: 0.44–1.10). Having severe physical limitations (compared to no limitations) was associated with a 51–68% increase in dementia incidence rates within hospitalisations (IRR = 1.68, 95%CI: 1.48–1.88), the ACFI (IRR = 1.51, 95%CI: 1.30–1.72) and death certificates (IRR = 1.56, 95%CI: 1.19–1.93) but was not significantly related to dementia incidence rates when using pharmaceutical claims (IRR = 0.92, 95%CI: 0.74–1.11) or aged care assessments (IRR = 1.05, 95%CI: 0.86–1.25).

However, there were several variables that were consistently associated with increased dementia incidence rates: high levels of psychological distress, low BMI, insufficient physical activity and history of falls. A Parkinson’s diagnosis was related to a three to five times increase in dementia incidence rates across all datasets and self-reported poor memory was related to a 5 to 10-fold increase in dementia incidence rates across all datasets.

The median survival from first dementia diagnosis was 2.7 years (95%CI: 2.61–2.78) (excluding 110 cases detected only on death certificates). Figure 4 shows that this varied significantly by first source of dementia diagnosis with cases detected first via medications surviving a median of 3.74 years (95%CI: 3.66–3.83) compared to 2.98 years for aged care assessments (95%CI: 2.84–3.15); 1.80 years for the ACFI (95%CI: 1.70–1.95) and 1.98 years for those first flagged in hospitalisations (95%CI: 1.82–2.09). Adjusted Hazard Ratios (AdjHR) controlling for age and sex, show that the differences in survival remained (see Table 4) with cases detected first within the ACFI and hospitalisations dying at two and a half times the rate of those detected first within pharmaceutical claims (ACFI AdjHR = 2.46; 95%CI: 2.16–2.79; hospitalisations AdjHR = 2.44; 95%CI: 2.19–2.72); and within assessments at one and half times the rate (AdjHR = 1.52; 95%CI: 1.35–1.70).

**Figure 6:**
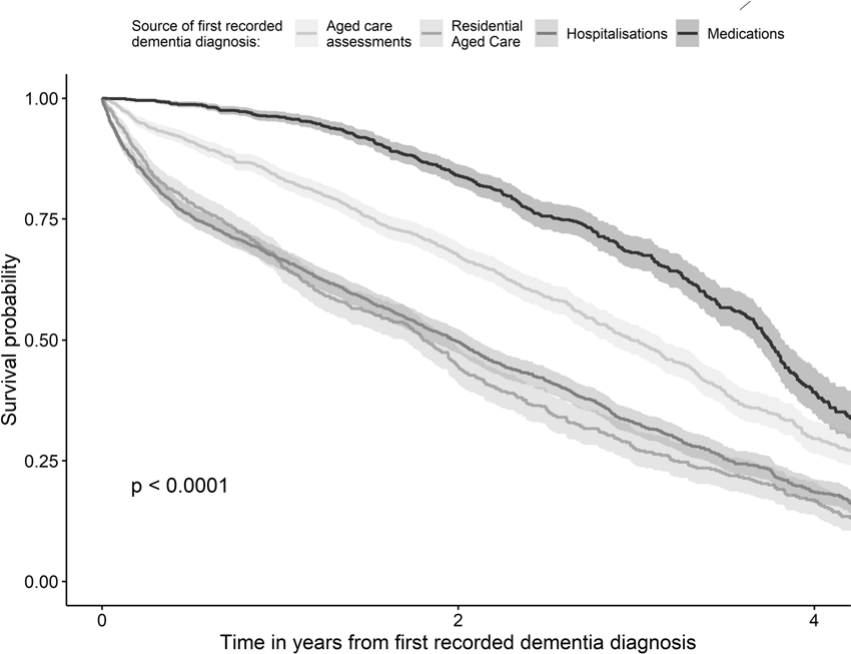
Survival from first recorded dementia diagnosis by source of first recorded dementia diagnosis. As dementia-specific medications are generally indicated only for mild-moderate dementia, cases detected in this dataset are picked up earlier in the disease course resulting in a longer survival time from first diagnosis until death compared to other data sources.

**Table 4:** Survival following a dementia diagnosis by source of first dementia diagnosis.

Source of first dementia diagnosis	N	Unadjusted median survival Years (95%CI)	Adjusted* HR (95%CI)	p value
Pharmaceutical claims	1416	3.74 (3.66-3.83)	*reference*	
Aged care assessments	1546	2.98 (2.84-3.15)	1.52 (1.35-1.70)	<0.001
Hospitalisations	1973	1.98 (1.82-2.09)	2.44 (2.19-2.72)	<0.001
ACFI	900	1.80 (1.70-1.95)	2.46 (2.16-2.79)	<0.001

NOTE: excludes 110 cases which were identified through death certificates only.

*Adjusted for age and sex.

## Discussion

Measuring dementia incidence in a population or cohort study is difficult for three main reasons: (i) dementia remains undiagnosed in the early stages^6^; (ii) while prevalence in the oldest old may be quite high, prevalence in the total population is low, meaning the sample size required is large^21^; and (iii) persons living with dementia may be disproportionately missed from surveys or follow-up due to their cognitive deficits^3^. Routinely collected administrative data have the potential to address at least the last two barriers as they are available at scale and can be linked to baseline data even if a person has been lost to follow-up.

Our study has extended that of Waller *et al*.^5^ by showing that dementia incidence rates derived using multiple routinely collected Australian administrative data mirror the age-specific patterns found in most major cohort studies^22–24^. With the exception of the oldest age category, the rates doubled approximately every 6–7 years which is in line with the pattern found by others^23,25^.

Compared to a recent meta-analysis of global dementia incidence studies^22^, our incidence rate estimates were lower across all ages. This may reflect both under-ascertainment of dementia in administrative data and a ‘healthy cohort effect’ in the 45 and Up Study (which had a response rate of 18%^26^). Nonetheless, our incidence rate estimates for the 80–84- and 85–89-year age groups reached greater than 90% of the published global rates. The Sydney Older Persons study provides one relatively recent Australian comparison and similarly shows slightly higher incidence rates to the current study, although it is based on a small sample^27^. The rates observed in the current study are consistent with other studies internationally using administrative data^28^.

We systematically examined the five main sources of administrative data currently available in Australia that can be used to identify dementia. We showed clear differences between the dementia populations that were identified in each source. It was also clear from examining survival data that individuals interact with different services (and therefore generate data within different administrative datasets) at different points along their trajectory of dementia progression.

Based on survival time, pharmaceutical claims appeared to occur early in disease progression. The most common claims for dementia-related medications are for cholinesterase inhibitors. In Australia, these medicines are approved only for mild to moderate dementia (as determined by testing with the Mini-mental State Evaluation)^29^ so by definition, people who are diagnosed at a more advanced state with a shorter life expectancy are not prescribed these medications.

Pharmaceutical claims were also more common among females, those from areas of least disadvantage and those with fewer comorbidities. Our findings suggest that there is reduced prescribing of dementia-specific medicines in the older age groups, and that cases flagged through medications alone are likely to not be representative of all dementia cases. Internationally, others have found that prescribing of cholinesterase inhibitors declines with age due to the increased presence of comorbidities and polypharmacy^30^. In Australia cost of medications may also have been a deterrent in low socio-economic areas and there have been barriers to prescribing cholinesterase inhibitors without access to specialist physicians (from whom confirmation of diagnosis was required to be eligible for subsidised cholinesterase medication), which may have resulted in inequitable access and could explain higher levels of prescribing in areas of least disadvantage^31^. Similar disparities have been reported in the United Kingdom^32^.

Aged care assessments appeared to detect cases earlier than both the Aged Care Funding Instrument and hospitalisations, although not as early as pharmaceutical claims. There were also some key demographic differences between these datasets. Males were more likely to be detected through a hospitalisation – likely due to the increased propensity of males to be hospitalised in older ages^33^. Females and those who were not married or partnered were more likely to be detected through the ACFI whereas males and those who were married/partnered were more likely to be detected within the aged care assessment program. This reflects the differing likelihood of being cared for at home rather than within residential aged care (nursing home) if a co-resident partner is available, as well as the decreased likelihood that a female will have an available carer at home, due to greater female longevity^10^.

We examined the association between dementia incidence rates and a range of established risk factors which illustrates the potential impact of the differences in dataset coverage outlined above. Females have a greater lifetime risk of developing dementia^34^. However, this seems to be mostly due to overall longevity and increased survival with dementia^35^ rather than an increased rate of dementia occurrence^36^. Major cohort studies of dementia incidence have had mixed findings in relation to gender differences, with many showing no differences^34,36–38^, some reporting higher incidence rates in females, particularly in the oldest ages^39^ and others higher incidence rates in males^40^. Our study showed differences between the sexes dependent on the data source used, with males having significantly higher incidence rates using hospitalisations or death certificates and females having higher incidence rates when using the ACFI. The choice of datasets will therefore influence the strength and the direction of this relationship.

Diabetes in mid-life is well established as a risk factor for dementia^41^. However, the relative risk is higher for vascular dementia compared to Alzheimer’s disease^42^, while cholinesterase inhibitors are at present only indicated for the latter. Additionally, given the complexities and medications involved with managing diabetes, it is related to lower prescribing of non-diabetic medications such as cholinesterase inhibitors^30^. This results in an inverse relationship between diabetes and dementia as defined by pharmaceutical claims alone, but a more expected predictive relationship between diabetes and subsequent increased dementia incidence rates in all other datasets.

Being married has been found to be protective against dementia, while being single or widowed is a risk factor^43^. This is thought to be due to an increased propensity to engage in healthier lifestyle behaviours and have increased levels of social interaction among those who are partnered. But marital status is also related to an individual’s pattern of interaction with health care and particularly with social care services^44^. Using pharmaceutical claims alone to detect dementia results in an apparent ‘protective’ effect of being single or widowed/divorced, whereas using the ACFI alone results in the opposite finding with being married or partnered as protective.

Similarly, larger social networks have been found to be protective for dementia^45^ which aligns with the current findings (within aged care assessments, hospitalisations, the ACFI and death certificates) of reduced dementia incidence rates among those reporting higher numbers of people that can be depended upon. However, the opposite relationship was found within pharmaceutical claims.

It is possible that people with partners or larger support networks are more likely to be prescribed medications for dementia for several reasons. They may be diagnosed at an earlier stage of disease progression and therefore are more likely to be eligible for treatment. It may also be that the partners or family of a person living with dementia seek to initiate treatment rather than the patient themselves or that the prescribing physician is more confident in medication adherence due to the presence of family or other carers. This is supported by findings that being married is predictive of pharmaceutical treatment for dementia^43,46^.

Despite these inconsistencies there were also factors that were significantly related to elevated dementia incidence rates within all datasets. These included low BMI, insufficient physical activity, higher levels of psychological distress at baseline, a history of falls, Parkinson’s disease and self-reported poor memory. Dementia is known to be strongly associated with Parkinson’s disease, with 40% of people with Parkinson’s also living with dementia^47^. Physical activity is also well-established as a protective factor for dementia^48^. Low BMI, psychological distress, history of falls and self-reported poor memory have all been identified as possible indicators of pre-clinical dementia^49–52^, and their relationship in the current study supports these findings. It is important to note that due to the relatively short follow-up from baseline to dementia ascertainment we were not seeking to identify causal factors in the current study but to examine factors associated with the recording of dementia in administrative data. For example, Kivimaki *et al*.^53^ elegantly demonstrated the importance of temporal distance when examining the relationship between BMI and dementia and the current findings are consistent with a decline in BMI that occurs prior to diagnosis and we do not suggest that high BMI is protective for dementia.

As primary care with specialist referral is the most likely path to first dementia diagnosis^54^, the availability of diagnoses from within primary or outpatient specialist care settings would permit much more complete case ascertainment, particularly in younger ages. This data gap in Australia could be remedied through the introduction of “My Health Record” which provides an electronic personally controlled single health summary for Australian patients across primary and secondary care^55^, but is not yet available for secondary analyses. Other platforms, such as “Medicine Insight”^56^ which extract data directly from primary care practices could also provide diagnosis information, but at present the available data are practice rather than population-based. There is also work underway to construct a national dementia register^57^. Other countries such as Sweden have shown the potential benefit of such registries^58^, but it is likely to be some time before this becomes a useful resource for monitoring dementia in the Australian population.

The major strengths of this study include the large sample size and inclusion of five separate administrative health data sources to detect dementia. Additionally, this study investigated dementia incidence rates using linked data across a wide range of ages and for both sexes which has not to our knowledge been done before in Australia. Although the low response rate in the 45 and Up Study reduces its utility for generating incidence rate estimates, findings based on comparing groups within the cohort are generalisable to the broader population^59^. The inclusion of a range of participant characteristics in the 45 and Up Study baseline survey allowed us to comprehensively investigate potential biases across data sources in a depth not previously possible. The main limitations include the lack of available primary care diagnoses and information regarding initial dementia onset and disease severity. We acknowledge that there was no way of assessing the administrative data against a gold standard clinical assessment of dementia to ascertain the true date of dementia onset. This would have allowed a much more in-depth exploration of sensitivity and positive predictive values. We attempted to fill this gap by examining dementia survival as a proxy for disease progression. Finally, we also note that there is evidence from the United Kingdom that recording of dementia in some administrative data collections appears to be improving over time^60,11^. While this hasn’t been investigated in depth in Australia it is likely that recording of diagnoses in hospitalisations and death certificates has also improved over time. This may mean that timeframe for recording of dementia as well as data source should be considered when assessing outcomes.

## Conclusions

Multiple linked Australian administrative data sources provide reasonable estimates of dementia incidence rates that mirror the age-specific patterns found within other major cohort studies. Relative coverage appears very high in those aged in their eighties (over 90% compared to global rates) but is slightly poorer in both the younger age groups where interaction with the aged care system is lower, and in the oldest age groups where use of dementia-specific medicines is less common. People identified with dementia in different administrative datasets have different characteristics, reflecting the factors that drive interaction with specific services, and suggesting that bias may be introduced if single data sources are used to measure outcomes. In randomised controlled trials this is likely to be irrelevant as the bias should be random but nevertheless using multiple datasets will increase study power due to the increased capture of cases. For cohort studies the potential for bias is non-trivial and multiple data sources should be used where possible. Variables of interest should be examined carefully to ascertain whether they could be related to the propensity for identification of dementia in specific datasets. Checking for consistency of relationships across data sources may be one method of providing reassurance that bias does not exist.

## Methods

### Setting and design

This was a prospective observational data linkage study. It was part of the “Exploring the relationship between Social care, primary and secondary Health service use and adverse health OUTcomes (SHOut)” project which draws data from the Sax Institute’s 45 and Up Study^26^, a prospective cohort of 267,153 people in New South Wales (NSW), Australia. Recruitment to the 45 and Up study was in 2006–2009 via random sampling from the Department of Human Services (formerly Medicare) enrolment database, Australia’s national universal insurance provider. Participants joined by completing a self-administered questionnaire and provided written consent to long-term follow-up including linkage with administrative health datasets.

Data from the 45 and Up baseline survey were linked to administrative datasets as illustrated in Fig. 5.

**Figure 7:**
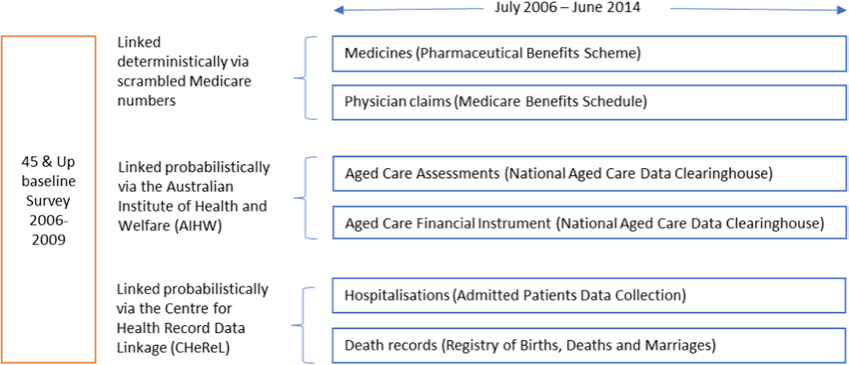
Data Linkage schema depicting the routinely collected administrative datasets and how each was linked to the baseline survey. Data from the Pharmaceutical Benefits Scheme and Medicare Benefits Schedule were provided by the Department of Human Services; Information regarding the Centre for Health Record Linkage can be found at http://www.cherel.org.au.

### Identifying the study cohort

All participants who completed the 45 and Up Study baseline survey and were alive at study commencement (two years following baseline) were eligible for entry to this study. Participants were excluded if they held a Department of Veteran’s Affairs health insurance card due to incomplete pharmaceutical claims data or if there were likely data linkage errors. In order to ensure the cohort were dementia-free at baseline, people were excluded if they had a dementia diagnosis recorded within any of the five administrative datasets within two years of their baseline survey based on the algorithm defined below.

### Defining dementia

Dementia was defined based on: (i) any pharmaceutical claim for dementia-specific medicines; or dementia diagnosis codes using previously defined algorithms^5,61^ in either: (ii) hospitalisations; (iii) aged care assessments; (iv) the Aged Care Funding Instrument or (v) underlying or contributing cause of death on death certificates (see Supplementary table S2).

Hospitalisations include all inpatient episodes but do not include emergency department visits where no admission occurred and do not include outpatient visits. Aged care assessments are conducted to assess eligibility for government subsidised home and community-based support or residential aged care in Australia. They include a face to face assessment by a trained aged care assessment team and include detailed assessment of independence as well as recording multiple health conditions supported by documented clinical diagnoses from a geriatrician, GP or another specialist. The Aged Care Funding Instrument (ACFI) is used by trained assessors as a way of establishing the level of care needed by a resident once they have entered a residential aged care facility^62^. Further detail regarding each type of aged care assessment can be found within the relevant guidelines^63^ and aged care staff making assessments must adhere to legislated standards set out in the Quality of Care Principles 2014^64^.

### Outcome measures

The main outcomes used to address each of the aims were: (i) age-specific dementia incidence rates; (ii) age-adjusted dementia incidence rate ratios; (iii) survival time from first dementia diagnosis until death. These were calculated separately based on dementia diagnoses within each of five administrative data sources and using a combination of the five datasets.

### Cohort characteristics

Characteristics of dementia cases were examined using self-reported data from the 45 and Up Study baseline survey which included socio-demographic variables, health risk factors, health status and self-reported chronic conditions as defined in Supplementary table S3. Missing data on survey variables were treated as separate categories for descriptive analyses and were excluded from calculation of incidence rate ratios.

### Statistical analyses

Dementia ‘cases’ were defined as the number of people with a dementia diagnosis detected within the follow-up period from study entry (2 years after completion of the 45 and Up Study baseline survey: 2008–2011) to study end (30 June 2014). Dementia incidence rates were calculated as the number of cases divided by the number of person years of follow-up measured from study entry to first dementia flag, death or study end, whichever came first. Age-specific dementia incidence rates were calculated using 5-year age groups based on age at study entry and age-standardised dementia incidence rates were calculated using direct standardisation to the New South Wales standard population for June 2011 based on single year of age.

Age-adjusted incidence rate ratios (IRRs) were calculated to examine selected baseline characteristics and dementia incidence rates across the different datasets.

Kaplan Meier curves and Cox proportional hazards regression were used to assess the relationship between source of first dementia flag and survival adjusted for age and sex.

Data management was carried out using SAS software. SAS and all other SAS Institute Inc. product or service names are registered trademarks or trademarks of SAS Institute Inc., Cary, NC, USA. Data analysis was carried out using R version 6.0^65^.

### Ethical approval

This study was conducted in accordance with the Australian National Health and Medical Research Council’s National Statement on Ethical Conduct in Human Research^66^. Approval for the 45 and Up Study was granted by the University of New South Wales Human Research Ethics committee and for the SHOut study by the NSW Population and Health Services, Aboriginal Health and Medical Research Council of NSW, Department of Veteran’s Affairs and the AIHW research ethics committees.

## Supplementary Material

Supplementary Information

## Data Availability

The 45 and Up Study dataset is available under licence from the Sax Institute. For further information regarding the process for accessing and linking the administrative datasets please refer to http://www.cherel.org.au/.
