# Supplementary material for: Measuring dementia incidence within a cohort of 267,153 older Australians using routinely collected linked administrative data
Source: Sci Rep. 2020 May 29;10:8781. doi: 10.1038/s41598-020-65273-w (PMC7260191; doi:10.1038/s41598-020-65273-w)
Supplement: Supplementary file 1 — Supplementary Information [file 41598_2020_65273_MOESM1_ESM.docx]

**Supplementary Material**

**Table S1: Age-adjusted Incidence Rate Ratios for selected characteristics by administrative dataset**

|  | **Medications** | **Aged care assessments** | **Hospitalisations** | **Residential Aged Care Funding Instrument** | **Death Certificates** |
| --- | --- | --- | --- | --- | --- |
| **Characteristic** | **Adj IRR**  **(95% CI)** | **Adj IRR**  **(95% CI)** | **Adj IRR**  **(95% CI)** | **Adj IRR**  **(95% CI)** | **Adj IRR**  **(95% CI)** |
| Sex (ref = female) |  |  |  |  |  |
| Male | 0.93  ( 0.83 - 1.03 ) | 1.09  ( 1.01 - 1.17 ) | 1.16  ( 1.08 - 1.24 ) | 0.92  ( 0.83 – 1.00 ) | 1.26  ( 1.11 - 1.42 ) |
| Marital status (ref=Married/Partner) |  |  |  |  |  |
| Single | 0.61  ( 0.32 - 0.91 ) | 1.03  ( 0.84 - 1.22 ) | 1.24  ( 1.07 - 1.4 ) | 1.77  ( 1.6 - 1.94 ) | 1.31  ( 1 - 1.62 ) |
| Widowed/divorced/ separated | 0.82  ( 0.7 - 0.93 ) | 1.00  ( 0.91 - 1.09 ) | 1.04  ( 0.95 - 1.13 ) | 1.33  ( 1.23 - 1.42 ) | 1.02  ( 0.84 - 1.19 ) |
| Education (ref = University or higher) |  |  |  |  |  |
| Did not complete school | 1.03  ( 0.88 - 1.18 ) | 1.1  ( 0.97 - 1.23 ) | 1.16  ( 1.03 - 1.29 ) | 1.14  ( 1.01 - 1.28 ) | 1.29  ( 1.04 - 1.54 ) |
| High school/ trade | 1.08  ( 0.93 - 1.23 ) | 1.10  ( 0.96 - 1.23 ) | 1.06  ( 0.93 - 1.19 ) | 1.00  ( 0.86 - 1.14 ) | 1.12  ( 0.86 - 1.38 ) |
| Income (ref= $70,000+) |  |  |  |  |  |
| <$20,000 | 1.08  ( 0.83 - 1.32 ) | 1.30  ( 1.05 - 1.56 ) | 1.63  ( 1.41 - 1.85 ) | 1.43  ( 1.17 - 1.7 ) | 1.38  ( 0.84 - 1.92 ) |
| $20,000-$49,999 | 1.19  ( 0.94 - 1.44 ) | 1.14  ( 0.88 - 1.4 ) | 1.29  ( 1.06 - 1.53 ) | 1.10  ( 0.82 - 1.37 ) | 1.16  ( 0.6 - 1.72 ) |
| $50,000-69,999 | 1.2  ( 0.88 - 1.53 ) | 1.08  ( 0.76 - 1.4 ) | 1.21  ( 0.9 - 1.52 ) | 0.91  ( 0.56 - 1.27 ) | 0.98  ( 0.31 - 1.65 ) |
| Not specified | 1.36  ( 1.11 - 1.61 ) | 1.34  ( 1.08 - 1.6 ) | 1.69  ( 1.46 - 1.92 ) | 1.37  ( 1.1 - 1.64 ) | 1.45  ( 0.9 - 2.01 ) |
| Remoteness area (ref = Major cities) |  |  |  |  |  |
| Inner Regional | 0.92  ( 0.81 - 1.03 ) | 0.84  ( 0.75 - 0.94 ) | 0.99  ( 0.9 - 1.09 ) | 1.03  ( 0.93 - 1.12 ) | 0.91  ( 0.73 - 1.09 ) |
| Outer Regional/Remote/ V Remote | 0.77  ( 0.58 - 0.95 ) | 0.78  ( 0.61 - 0.96 ) | 1.01  ( 0.86 - 1.17 ) | 0.96  ( 0.79 - 1.14 ) | 0.81  ( 0.51 - 1.12 ) |
| Disadvantage (ref = Q5 Least disadvantage) |  |  |  |  |  |
| Q1 Most disadvantaged | 0.83  ( 0.69 - 0.98 ) | 1.16  ( 1.04 - 1.29 ) | 1.45  ( 1.32 - 1.57 ) | 1.42  ( 1.29 - 1.55 ) | 1.32  ( 1.09 - 1.56 ) |
| Q2 | 0.89  ( 0.74 - 1.04 ) | 1.09  ( 0.96 - 1.22 ) | 1.29  ( 1.16 - 1.42 ) | 1.23  ( 1.09 - 1.37 ) | 1.29  ( 1.05 - 1.54 ) |
| Q3 | 0.88  ( 0.72 - 1.03 ) | 1.14  ( 1 - 1.27 ) | 1.17  ( 1.03 - 1.3 ) | 1.15  ( 1.01 - 1.3 ) | 1.03  ( 0.77 - 1.29 ) |
| Q4 | 0.92  ( 0.76 - 1.08 ) | 1.14  ( 1.01 - 1.28 ) | 1.22  ( 1.08 - 1.36 ) | 1.06  ( 0.91 - 1.21 ) | 1.31  ( 1.05 - 1.57 ) |
| Country of Birth (ref=Australia) |  |  |  |  |  |
| Overseas | 1.05  ( 0.94 - 1.15 ) | 1.02  ( 0.93 - 1.11 ) | 1.00  ( 0.91 - 1.09 ) | 0.93  ( 0.83 - 1.02 ) | 1.14  ( 0.97 - 1.3 ) |
| Language at home (ref = English) |  |  |  |  |  |
| Other | 0.97  ( 0.81 - 1.13 ) | 0.99  ( 0.86 - 1.12 ) | 1.06  ( 0.94 - 1.19 ) | 0.87  ( 0.73 - 1.01 ) | 1.14  ( 0.9 - 1.38 ) |
| Number of people can depend on (ref=Zero) |  |  |  |  |  |
| 1-4 | 1.25  ( 1 - 1.5 ) | 0.94  ( 0.75 - 1.12 ) | 0.83  ( 0.67 - 0.99 ) | 0.82  ( 0.63 - 1.01 ) | 0.87  ( 0.52 - 1.22 ) |
| 5-10 | 1.26  ( 1.01 - 1.52 ) | 0.80  ( 0.61 - 0.99 ) | 0.68  ( 0.51 - 0.84 ) | 0.64  ( 0.45 - 0.84 ) | 0.65  ( 0.29 - 1.01 ) |
| 11+ | 1.25  ( 0.98 - 1.53 ) | 0.68  ( 0.45 - 0.91 ) | 0.63  ( 0.41 - 0.85 ) | 0.53  ( 0.28 - 0.78 ) | 0.53  ( 0.09 - 0.97 ) |
| Smoking status (ref = Never smoked) |  |  |  |  |  |
| Past smoker | 0.91  ( 0.8 - 1.01 ) | 1.07  ( 0.98 - 1.16 ) | 1.09  ( 1.01 - 1.18 ) | 1.00  ( 0.9 - 1.09 ) | 1.03  ( 0.86 - 1.2 ) |
| Current smoker | 0.77  ( 0.44 - 1.1 ) | 1.61  ( 1.26 - 1.96 ) | 1.74  ( 1.47 - 2 ) | 1.62  ( 1.28 - 1.95 ) | 1.66  ( 1.09 - 2.23 ) |
| Number of drinks per week (ref = Zero) |  |  |  |  |  |
| 1-4 drinks | 1.04  ( 0.9 - 1.18 ) | 0.90  ( 0.79 - 1.02 ) | 0.84  ( 0.72 - 0.96 ) | 0.85  ( 0.72 - 0.97 ) | 0.87  ( 0.66 - 1.09 ) |
| 5-7 drinks | 1.29  ( 1.15 - 1.43 ) | 1.03  ( 0.91 - 1.15 ) | 0.89  ( 0.78 - 1.01 ) | 0.94  ( 0.82 - 1.07 ) | 0.96  ( 0.74 - 1.19 ) |
| 8-14 drinks | 1.07  ( 0.91 - 1.22 ) | 0.9  ( 0.76 - 1.04 ) | 0.83  ( 0.69 - 0.96 ) | 0.79  ( 0.64 - 0.94 ) | 0.75  ( 0.47 - 1.03 ) |
| 15 or more | 0.94  ( 0.75 - 1.12 ) | 0.99  ( 0.8 - 1.18 ) | 0.9  ( 0.72 - 1.09 ) | 0.78  ( 0.57 - 0.99 ) | 0.88  ( 0.48 - 1.28 ) |
| BMI category (ref = Healthy weight) |  |  |  |  |  |
| Underweight | 1.24  ( 1.09 - 1.39 ) | 1.46  ( 1.35 - 1.57 ) | 1.38  ( 1.27 - 1.49 ) | 1.48  ( 1.36 - 1.59 ) | 1.34  ( 1.14 - 1.55 ) |
| Overweight | 0.75  ( 0.64 - 0.86 ) | 0.79  ( 0.69 - 0.89 ) | 0.83  ( 0.73 - 0.93 ) | 0.82  ( 0.71 - 0.92 ) | 0.72  ( 0.52 - 0.93 ) |
| Obese | 0.52  ( 0.34 - 0.7 ) | 0.75  ( 0.57 - 0.92 ) | 0.87  ( 0.72 - 1.02 ) | 0.92  ( 0.75 - 1.09 ) | 0.98  ( 0.64 - 1.32 ) |
| Physical activity (ref=Meets guidelines) |  |  |  |  |  |
| Does not meet guidelines | 1.19  ( 1.09 - 1.28 ) | 1.4  ( 1.31 - 1.48 ) | 1.52  ( 1.43 - 1.6 ) | 1.61  ( 1.52 - 1.7 ) | 1.58  ( 1.42 - 1.74 ) |
| Physical functioning (ref=No Limitations) |  |  |  |  |  |
| Minor Limitation | 0.99  ( 0.8 - 1.18 ) | 0.85  ( 0.61 - 1.08 ) | 0.86  ( 0.61 - 1.1 ) | 0.87  ( 0.61 - 1.13 ) | 0.89  ( 0.38 - 1.4 ) |
| Moderate Limitation | 0.9  ( 0.73 - 1.08 ) | 0.87  ( 0.67 - 1.07 ) | 1.01  ( 0.81 - 1.22 ) | 0.95  ( 0.73 - 1.17 ) | 0.94  ( 0.55 - 1.33 ) |
| Severe Limitation | 0.92  ( 0.74 - 1.11 ) | 1.05  ( 0.86 - 1.25 ) | 1.68  ( 1.48 - 1.88 ) | 1.51  ( 1.3 - 1.72 ) | 1.56  ( 1.19 - 1.93 ) |
| Psychological distress (ref= Low) |  |  |  |  |  |
| Moderate | 1.52  ( 1.37 - 1.67 ) | 1.35  ( 1.23 - 1.48 ) | 1.56  ( 1.44 - 1.67 ) | 1.49  ( 1.36 - 1.62 ) | 1.4  ( 1.16 - 1.64 ) |
| High | 1.46  ( 1.2 - 1.72 ) | 1.94  ( 1.74 - 2.13 ) | 2.08  ( 1.91 - 2.25 ) | 1.82  ( 1.63 - 2.02 ) | 2.73  ( 2.42 - 3.04 ) |
| Very High | 1.70  ( 1.27 - 2.13 ) | 1.33  ( 1.02 - 1.65 ) | 2.19  ( 1.91 - 2.46 ) | 1.50  ( 1.17 - 1.83 ) | 1.45  ( 0.75 - 2.15 ) |
| Diabetes (ref = No Diabetes) |  |  |  |  |  |
| Diabetes | 0.82  ( 0.67 - 0.98 ) | 1.12  ( 1.00 - 1.24 ) | 1.37  ( 1.26 - 1.47 ) | 1.25  ( 1.13 - 1.37 ) | 1.40  ( 1.18 - 1.62 ) |
| History of stroke (ref = No Stroke) |  |  |  |  |  |
| Stroke | 1.24  ( 1.00 - 1.47 ) | 1.52  ( 1.37 - 1.66 ) | 1.73  ( 1.60 - 1.87 ) | 1.47  ( 1.33 - 1.60 ) | 1.53  ( 1.29 - 1.78 ) |
| Parkinson's disease (ref=No Parkinson's) |  |  |  |  |  |
| Parkinsons | 4.46  ( 4.15 - 4.77 ) | 3.56  ( 3.31 - 3.81 ) | 4.22  ( 4.02 - 4.42 ) | 5.1  ( 4.79 - 5.42 ) | 4.12  ( 3.63 - 4.62 ) |
| Depression/anxiety (ref=No depression/anxiety) |  |  |  |  |  |
| depression/anxiety | 1.33  ( 1.19 - 1.47 ) | 1.32  ( 1.20 - 1.44 ) | 1.37  ( 1.26 - 1.49 ) | 1.24  ( 1.11 - 1.37 ) | 1.17  ( 0.93 - 1.42 ) |
| Heart disease (ref=No Heart Disease) |  |  |  |  |  |
| Heart disease | 0.87  ( 0.75 – 1.00) | 0.93  ( 0.83 - 1.02 ) | 1.01  ( 0.92 - 1.1 ) | 0.94  ( 0.84 - 1.04 ) | 1.04  ( 0.87 - 1.21 ) |
| Self-reported falls (ref=No falls) |  |  |  |  |  |
| Falls | 1.18  ( 1.07 - 1.29 ) | 1.33  ( 1.24 - 1.42 ) | 1.56  ( 1.47 - 1.64 ) | 1.55  ( 1.46 - 1.64 ) | 1.62  ( 1.46 - 1.78 ) |
| Self-reported memory (ref=Excellent) |  |  |  |  |  |
| Very good | 1.76  ( 1.42 - 2.09 ) | 1.11  ( 0.89 - 1.33 ) | 1.09  ( 0.89 - 1.28 ) | 1.03  ( 0.82 - 1.25 ) | 0.92  ( 0.52 - 1.32 ) |
| Good | 2.95  ( 2.64 - 3.27 ) | 1.68  ( 1.48 - 1.89 ) | 1.45  ( 1.27 - 1.63 ) | 1.61  ( 1.41 - 1.8 ) | 1.64  ( 1.28 – 2.00 ) |
| Fair | 6.35  ( 6.04 - 6.67 ) | 3.26  ( 3.06 - 3.46 ) | 2.72  ( 2.54 - 2.89 ) | 2.76  ( 2.57 - 2.96 ) | 3.37  ( 3.02 - 3.72 ) |
| Poor | 10.45  ( 10.09 - 10.81) | 6.49  ( 6.24 - 6.73 ) | 5.57  ( 5.35 - 5.79 ) | 5.21  ( 4.98 - 5.45 ) | 6.54  ( 6.12 - 6.95 ) |

**Table S2: Codes used to identify dementia within the administrative datasets**

| **Aged Care Assessment Program / Aged Care Funding Instrument**  - code recorded in an assessment | | |
| --- | --- | --- |
| **Condition codes** | | **Health condition** |
| **0500** | | Dementia in Alzheimer’s disease |
| **0501** | | Dementia in Alzheimer’s disease with early onset (<65 yrs) |
| **0502** | | Dementia in Alzheimer’s disease with late onset (>65 yrs) |
| **0503** | | Dementia in Alzheimer’s disease, atypical or mixed type |
| **0504** | | Dementia in Alzheimer’s disease, unspecified |
| **0510** | | Vascular dementia |
| **0511** | | Vascular dementia of acute onset |
| **0512** | | Multi-infarct dementia |
| **0513** | | Subcortical vascular dementia |
| **0514** | | Mixed cortical & subcortical vascular dementia |
| **0515** | | Other vascular dementia |
| **0516** | | Vascular dementia – unspecified |
| **0520** | | Dementia in other diseases classified elsewhere |
| **0521** | | Dementia in Pick’s disease |
| **0522** | | Dementia in Creutzfeldt-Jakob disease |
| **0523** | | Dementia in Huntington’s disease |
| **0524** | | Dementia in Parkinson’s disease |
| **0525** | | Dementia in human immunodeficiency virus (HIV) disease |
| **0526** | | Dementia in other specified diseases classified elsewhere |
| **0530** | | Other dementia |
| **0531** | | Alcoholic dementia |
| **0532** | | Unspecified dementia (includes presenile & senile dementia) |
| **Pharmaceutical Benefits Scheme**  - Any dispensing record | | |
| **ATC code** | **Drug name** | |
| **N06DA02** | Donepezil | |
| **N06DA03** | Rivastigmine | |
| **N06DA04** | Galantamine | |
| **N06DX01** | Memantine | |
| **Admitted Patient Data Collection/ Death Certificates – ICD10-AM**  - code recorded as principal or other diagnosis in APDC and as underlying or contributing cause of death on a death certificate | | |
| **Code** | **Description** | |
| **F00** | Dementia in Alzheimer's disease | |
| **F01** | Vascular dementia | |
| **F02** | Dementia in other diseases classified elsewhere | |
| **F03** | Unspecified dementia | |
| **F05.1** | Delirium superimposed on dementia | |
| **G30** | Alzheimer's disease | |
| **G31** | Other degenerative diseases of nervous system, not elsewhere classified | |

**Table S3: Covariates included as potential risk factors for dementia**

| **Factor Name** | **Levels** | **Data source** |
| --- | --- | --- |
| Age at study entry | Continuous | Calculated from 45 & Up Baseline reported date of birth + 2 years |
| Sex | “Male”, “Female” | 45 & Up baseline survey |
| Marital status | “Single”, “Married/Partner”, “Divorced/Separated/Widowed”, “Missing” | 45 & Up baseline survey |
| Highest Education Level | “Did not complete School”, “High School/ Trade”, “University or higher”, “Missing/Invalid” | 45 & Up baseline survey |
| Household income | “<$20,000”, “$20,000-$49,999”, “$50,000-$69,999”, “$70,000+”, “Not specified”, “Missing” | 45 & Up baseline survey |
| Remoteness area | Based on the ABS Australian Statistical Geographical Classification which defines locations based on the physical distance from an urban centre. Grouped as “Major cities”, “Inner regional”, “Outer regional/remote/ very remote”. | 45 & Up baseline survey |
| Quartile of Disadvantage | Based on the ABS Index of Relative Social Disadvantage – grouped by Quartiles: “Q1 – Most disadvantaged” – “Q5 – least disadvantaged”, “Missing” | 45 & Up baseline survey |
| Country of birth | “Australian Born”, “Born overseas”, “Missing” | 45 & Up baseline survey |
| Language Spoken at Home | “English”, “Other” | 45 & Up baseline survey |
| Number of people can depend on | Numeric – based on question: “How many people outside your house but within one hour of travel do you feel you can depend on or feel very close to?” | 45 & Up baseline survey |
| Smoking status | “Current smoker”, “Ex-smoker”, “Never smoked” | 45 & Up baseline survey |
| Number of alcoholic drinks per week | Based on the question “About how many alcoholic drinks do you have each week?” Coded to categories: “zero”, “1-4”, “5-7”, “8-14”, “15 or more” | 45 & Up baseline survey |
| Body Mass Index (BMI) category | Based on self-reported height and weight (BMI = W(kg)/H^2^(m)) coded to “Underweight (<20)”, “Normal weight (20-24.9)”, “Overweight (25-29.9)”, “Obese (30+)” | 45 & Up baseline survey |
| Physical Activity | Based on self-reported minutes of moderate and vigorous activity and coded as “Meets guidelines” and “Does not meet guidelines” with criteria of >=150 minutes of moderate activity or >=75 minutes of vigorous activity. | 45 & Up baseline survey |
| Self-reported physical functioning | “No limitations”, “Minor”, “Moderate”, “Severe”, “Missing” | 45 & Up baseline survey – Physical Functioning scale from the SF36 |
| Self-reported psychological distress | “Low Psychological distress”, “Moderate”, “High”, “Very high”, “Missing” | 45 & Up baseline survey – Psychological distress scale from the K10 |
| Self-reported diabetes | “Yes”, “No” – based on the question: “Has a doctor ever told you that you have …. Diabetes?” | 45 & Up baseline survey |
| Self-reported stroke | “Yes”, “No” – based on the question: “Has a doctor ever told you that you have …. Stroke?” | 45 & Up baseline survey |
| Self-reported heart disease | “Yes”, “No” – based on the question: “Has a doctor ever told you that you have …. Heart disease?” | 45 & Up baseline survey |
| Self-reported Parkinson’s disease | “Yes”, “No” – based on the question: “Has a doctor ever told you that you have …. Parkinson’s Disease?” | 45 & Up baseline survey |
| Self-reported depression or anxiety | “Yes”, “No” – based on the question: “Has a doctor ever told you that you have …. Depression/anxiety?” | 45 & Up baseline survey |
| Self-reported falls | Numeric – based on the question “During the past 12 months how many times have you fallen to the floor or ground?” coded to “yes” if had fallen once or more and “no” if not. | 45 & Up baseline survey |
| Self-reported memory | “Excellent”, “Very good”, “Good”, “Fair”, “Poor”, “Missing”, based on the question “In general, how would you rate your memory?” | 45 & Up baseline survey |
